# Supplementary material for: Paternal transmission of migration knowledge in a long-distance bird migrant
Source: Nat Commun. 2022 Mar 23;13:1566. doi: 10.1038/s41467-022-29300-w (PMC8943069; doi:10.1038/s41467-022-29300-w)
Supplement: Supplementary file 1 — Supplementary Information [file 41467_2022_29300_MOESM1_ESM.pdf]

Supplementary Table 1. Meta data on Finnish Caspian tern *Hydroprogne caspia* GPS-tracking events (n = 29) of birds belonging to family units (n = 8) during 2017-2019. Social state: The social state (female parent, male parent, foster male parent, young) and sex of tracked birds. Individuals highlighted in *italics* died before migration initiated and are not included in migration analyses. Individuals highlighted in different shades of grey are members of a parent-young pair investigated in breakup-analyses (see results). Migrating with: The parental bird in the family group migrating together with the young on its first outbound autumn migration. If the ID of the parental bird could not be proven directly from GPS-tracking, this is indicated by *italics* (cf. Fig. 1, Supplementary Fig. 1). Additional information on these cases: *Family 3:* There is no overlap in time and space between migration tracks of the young and the female parent (Supplementary Fig. 1). The male parent's GPS-device broke on the Egyptian wintering grounds 18-Nov 2017, i.e. there was no information available on the male's choice of migration route in 2018 (the year the young was tracked). However, since adults typically use the same migration routes in consecutive years (Fig. 3) and the migration track of the young in 2018 is an almost exact copy of its father's migration track in 2017 (Supplementary Fig. 1), the male parent was inferred to have guided the young. *Family 5:* There is no overlap in time and space between migration tracks of the young and the female parent (Supplementary Fig. 1). This leaves the male parent being the likely guide. In addition, both young initiated migrations simultaneously when leaving the breeding islet on 18-Aug 2018. *Family 8:* There is no overlap in time and space between migration tracks of the young and the female parent (Supplementary Fig. 1). This leaves the male parent being the likely guide. Migration initiates: Initiation of migration was defined as when the bird left the breeding islet not to return anymore for the season (Methods). Young 2 in Family 3 (highlighted with brackets around date) was deserted on the breeding islet by both parents (and its sib) and did not initiate migration in the real sense. It was predated by a white-tailed eagle (*Haliaetus albicilla*) on its night roost 5 km from the breeding islet on 22-Aug 2018, two days after leaving the breeding islet on its own. Distance (km), 1st outbound segment: Euclidian distance between breeding islet and first autumn migration stopover lasting > 1hr after migration initiated. Tag date: Date GPS-tag was deployed. Death/tag failure, Cause of death, Location: Information on death/tag failure. Status information is presented only until 31-December in the current year (the time window included in analyses). Cases marked with asterisks (*) were conformed in the field, the rest were deduced; w.-t. eagle = white-tailed eagle.

Family group	Social state	Migrating with	Migration initiates	Distance (km), 1st outbound segment	Tag date	Death/tag failure	Cause of death (* = confirmed)	Location
1	Young 1, female	Male parent	1-Aug 2017	94	5-Jul 2017	26-Oct 2017	Tag failure	Algeria
1	Male parent	-	1-Aug 2017	94	23-May 2017	-	-	-
1	Female parent	-	22-Jul 2017	159	25-May 2016	-	-	-
2	Young 1, male	Male parent	16-Jul 2017	80	5-Jul 2017	20-Jul 2017	Predated (goshawk)*	Finland
2	Male parent	-	16-Jul 2017	80	23-May 2017	-	-	-
2	Female parent	-	29-Jul 2017	107	22-May 2016	-	-	-
2	*Young 2, male*	-	-	-	*5-Jul 2017*	*9-Jul 2017*	*Predated (w.-t. eagle)**	*Finland*
3	Young 1, female	*Male parent*	14-Aug 2018	46	1-Aug 2018	-	-	-
3	Female parent	-	20-Aug 2018	272	24-May 2018	-	-	-
3	Young 2, male	-	(18-Aug 2018)	(5)	1-Aug 2018	22-Aug 2018	Predated (*w.-t.* eagle)*	Finland
4	Young 1, male	Male parent	21-Jul 2018	188	2-Jul 2018	20-Aug 2018	Predated (*w.-t.* eagle)*	Finland
4	Male parent	-	21-Jul 2018	188	24-May 2018	-	-	-
4	Female parent	-	30-Jul 2018	59	28-May 2018	-	-	-
4	*Young 2, male*	-	-	-	*9-Jul 2018*	*13-Jul 2018*	*Predated (otter)**	*Finland*
5	Young 1, female	*Male parent*	18-Aug 2018	88	1-Jul 2018	19-Oct 2018	Tag failure	Tunisia
5	Young 2, female	*Male parent*	18-Aug 2018	88	1-Jul 2018	22-Aug 2018	Predated (*w.-t.* eagle)*	Finland
5	Female parent	-	2-Aug 2018	88	23-May 2018	-	-	-
6	Young 1, male	Male parent	15-Jul 2018	74	28-Jun 2018	27-Dec 2018	Collision	Egypt
6	Male parent	-	15-Jul 2018	74	29-May 2017	-	-	-
6	Young 2, male	Female parent	29-Jul 2018	316	28-Jun 2018	-	-	-
6	Female parent	-	29-Jul 2018	316	28-May 2017	-	-	-
7	Young 1, female	Male parent	1-Aug 2018	91	3-Jul 2018	-	-	-
7	Male parent	-	1-Aug 2018	91	25-May 2018	-	-	-
7	Female parent	-	29-Jul 2018	73	23-May 2018	2-Oct 2018	Killed	Russia
7	*Young 2, male*	-	-	-	*3-Jul 2018*	*9-Jul 2018*	*Predated (w.-t. eagle)**	*Finland*
8	Young 1, male	*Male parent*	9-Aug 2019	74	23-Jul 2019	-	-	-
8	Young 2, male	Foster male parent	24-Aug 2019	73	12-Jul 2019	-	-	-
8	Foster male parent	-	24-Aug 2019	73	24-May 2018	-	-	-
8	Female parent	-	17-Jul 2019	26	24-May 2018	-	-	-

Supplementary Figure 1. Migration routes of Caspian terns (*Hydroprogne caspia*) belonging to separate family groups (n = 8) during the outbound autumn migration from Finland to Africa in 2017-2019. In family groups where all tracked young died/the GPS-tag failed *en route* (see Supplementary Table 1 for details) maps are scaled to approximately fit the data extent spatiotemporally, where ≥1 young survived until the end of the year maps are temporally delimited until December 31st the current year. Excluding one bird that was abandoned by its parents and died soon after (Young 2, Family 3), all other tracked individuals shown on maps (n = 25) successfully initiated their migration. If the map scale is unsuited for viewing all relevant information in one panel, smaller panels are inserted on top of the main panel.

Explanation of symbols:

/ / Migration route of GPS-tracked family group member. Colour indicates social status (see below). If the social status indicated in the legend inserted with the map is in *italics*, the identity of the guiding parent was deduced from indirect evidence (see Supplementary Table 1), not from GPS-tracking.

/ / Uniform symbol: Location of GPS-tracked bird when single (in broods of one) or latter young (in broods of two) in family group initiated migration. Split symbol: Location of GPS-tracked birds when single or latter young in family group initiated migration; number of segments corresponds to number of birds migrating together. Colour indicates social status. Date when migration started is given in text boxes with white background.

Place of death / location where tag failed of GPS-tracked bird. Background colour indicates social status. Date when mortality occurred is given in text boxes with grey background.

Location of adult bird and other guided young when non-guided chick died. Colour of outer band indicates social status.

/ Location of GPS-tracked bird by 31-December. Colour indicates social status, insert indicates location of guided young (applicable to Family 6 only).

Social status in family group (indicated by colour):

Female parent: **Red** (occurring as intact lines, large circles, large diamonds, combination symbols, place of death-symbol)

Male parent: **Light blue** (occurring as intact lines, large diamonds, combination symbols)

Foster male parent: **Dark blue** (occurring as intact lines, large diamonds, combination symbols)

Young 1: **Black** (occurring as small-large diamonds, combination symbols, place of death-symbol)

Young 2: **White** (occurring as small-large diamonds, combination symbols, hatched lines, place of death-symbols)

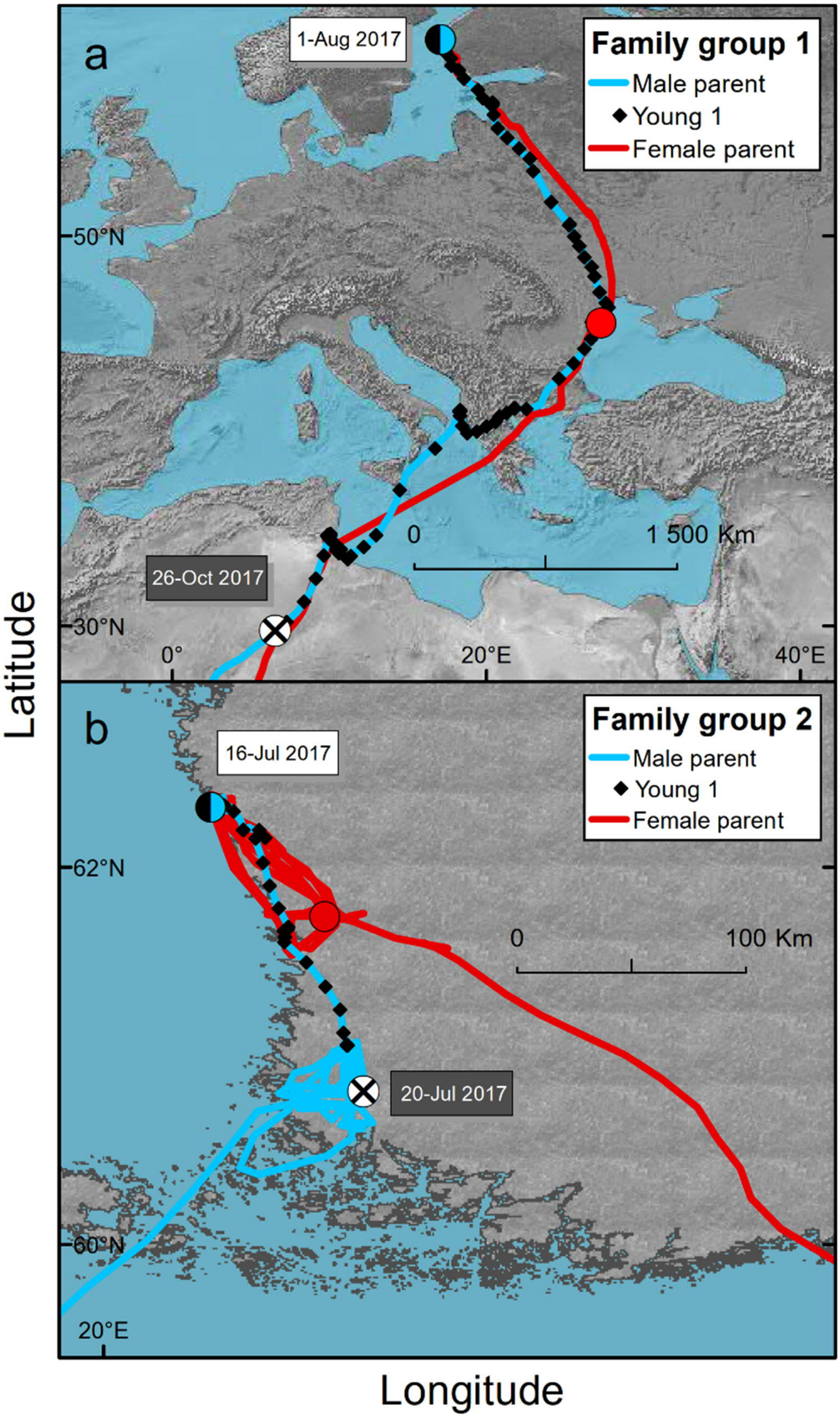
a
1-Aug 2017
Family group 1
Male parent
Young 1
Female parent
50°N
0
1 500 Km
26-Oct 2017
30°N
0°
20°E
40°E
b
16-Jul 2017
Family group 2
Male parent
Young 1
Female parent
62°N
0
100 Km
20-Jul 2017
60°N
20°E
Latitude
Longitude

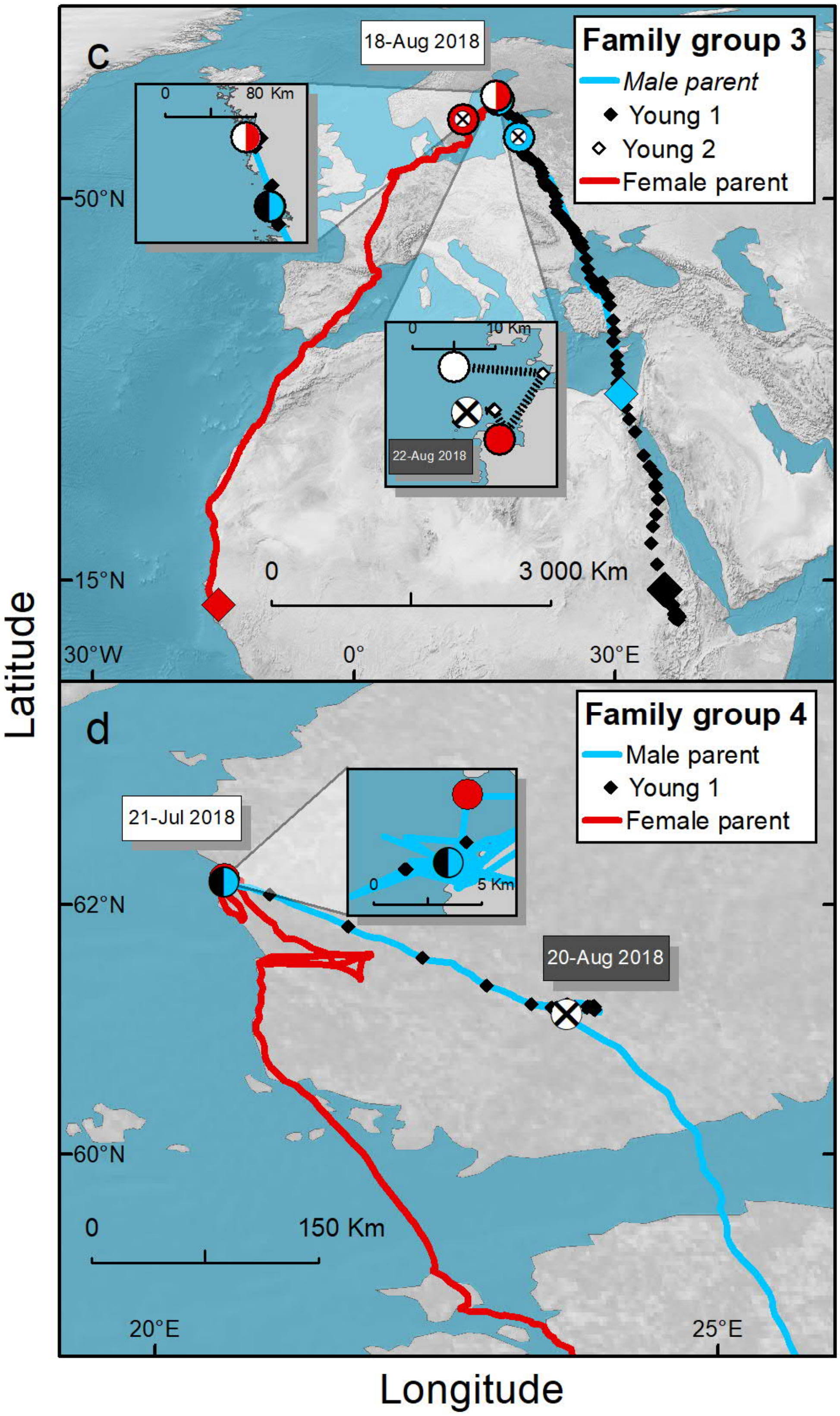
c
18-Aug 2018
Family group 3
Male parent
Young 1
Young 2
Female parent
0
80 Km
0
10 Km
22-Aug 2018
50°N
15°N
0
3 000 Km
30°W
0°
30°E
d
Family group 4
Male parent
Young 1
Female parent
21-Jul 2018
0
5 Km
62°N
20-Aug 2018
60°N
0
150 Km
20°E
25°E
Latitude
Longitude

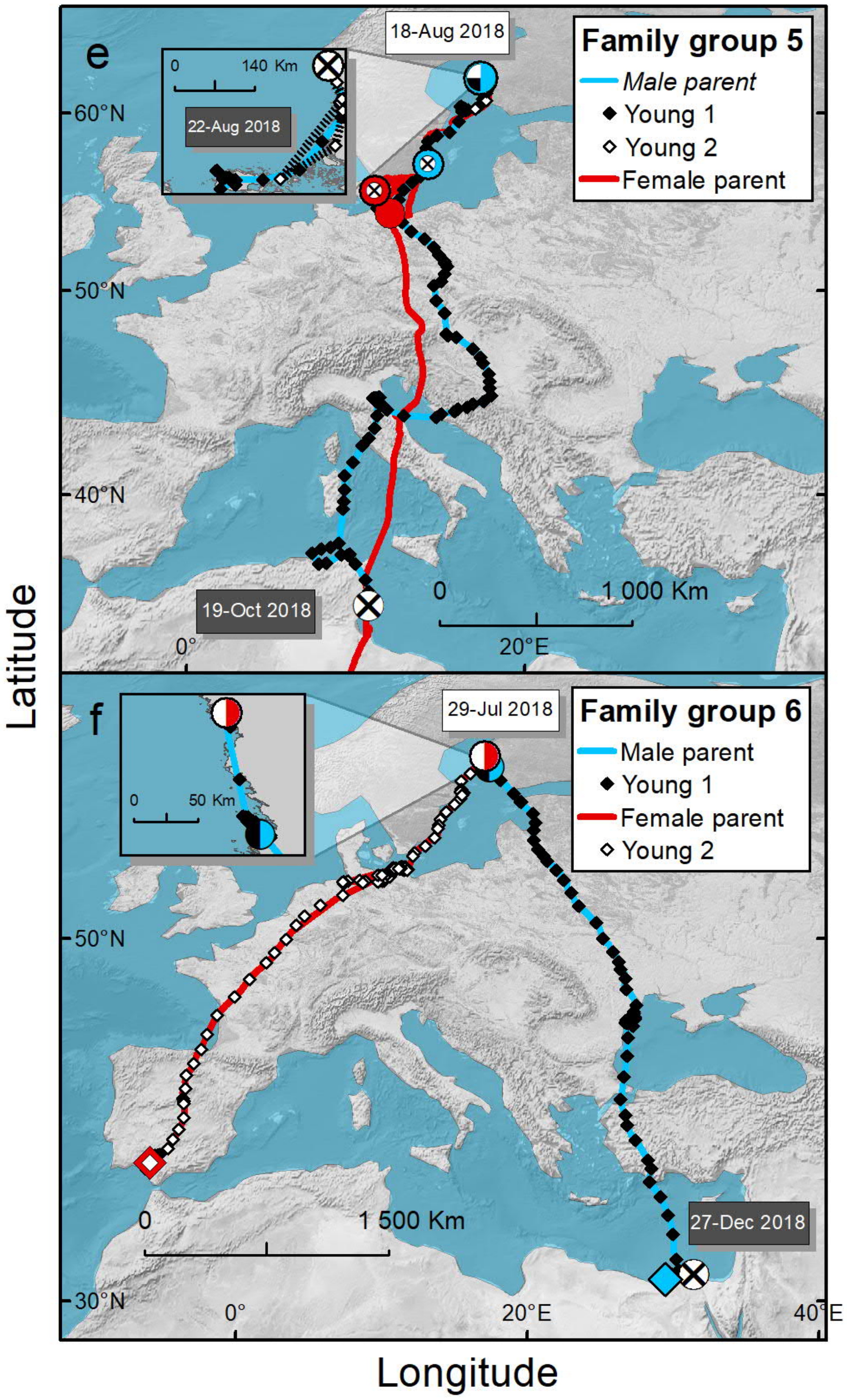
e
Family group 5
Male parent
Young 1
Young 2
Female parent
18-Aug 2018
0
140 Km
22-Aug 2018
19-Oct 2018
0
1 000 Km
60°N
50°N
40°N
0°
20°E
f
Family group 6
Male parent
Young 1
Female parent
Young 2
29-Jul 2018
0
50 Km
0
1 500 Km
27-Dec 2018
50°N
30°N
0°
20°E
40°E
Latitude
Longitude

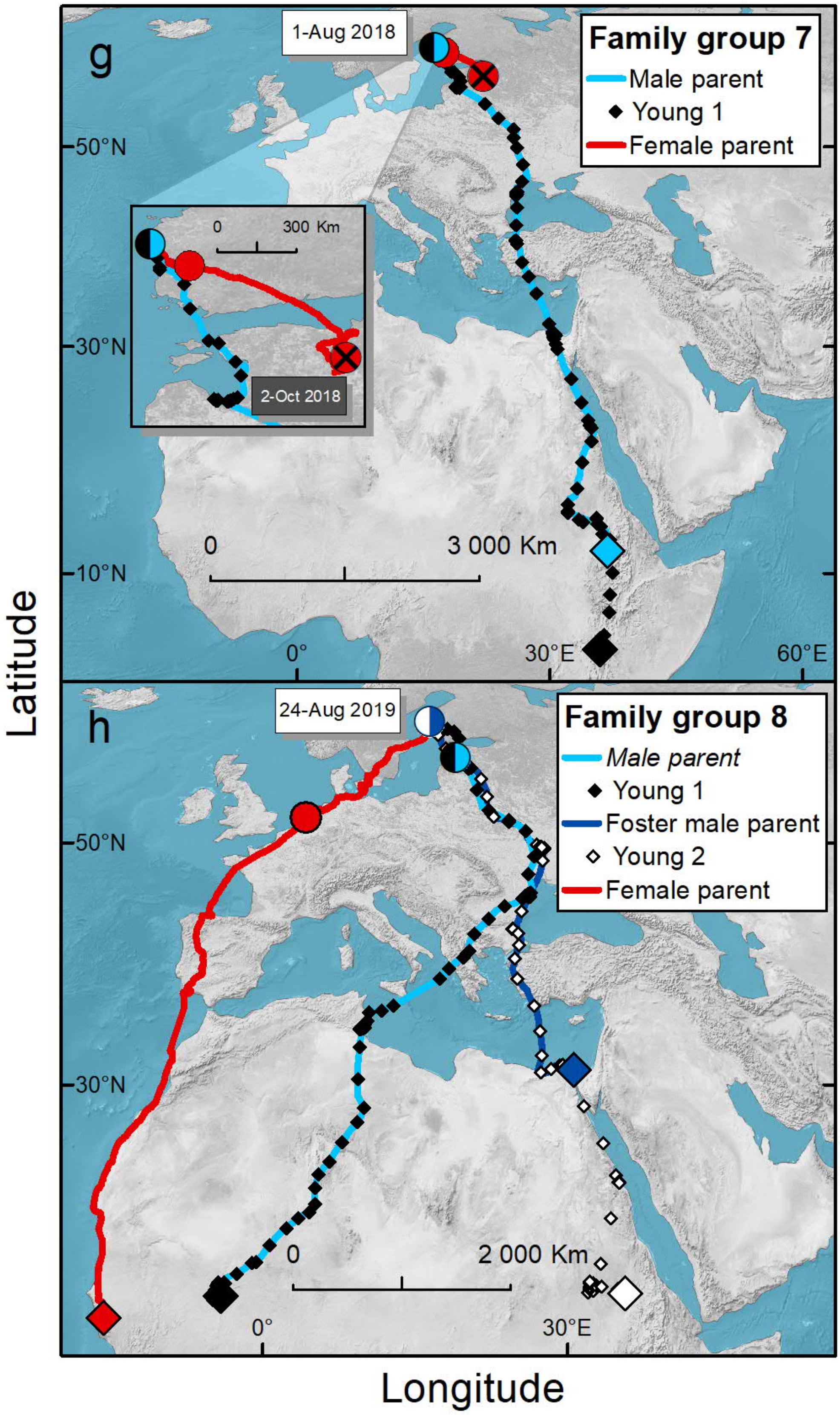
g
1-Aug 2018
Family group 7
Male parent
Young 1
Female parent
0
300 Km
2-Oct 2018
0
3 000 Km
50°N
30°N
10°N
0°
30°E
60°E
h
24-Aug 2019
Family group 8
Male parent
Young 1
Foster male parent
Young 2
Female parent
0
2 000 Km
50°N
30°N
0°
30°E
Latitude
Longitude

Panel-specific explanations:

Panel a: Migration tracks of terns belonging to Family unit 1 until failure of single young's (Young 1) GPS-tracker on 26-Oct 2017. Large circles indicate geographical locations of tracked birds when Young 1 and its parent initiated migration, geographical location of last received GPS-fix of Young 1.

Panel b: Migration tracks of terns belonging to Family unit 2 until death of single young (Young 1) on 20-Jul 2017. Large circles indicate geographical locations of tracked birds when Young 1 and its parent initiated migration, place of death of Young 1.

Panel c: Main panel: Migration tracks of terns belonging to Family unit 3 until 31-Dec 2018. Large circles indicate locations of tracked birds when latter young (Young 2) died. Large diamonds indicate wintering locations. Top-left insert panel: Family unit members' geographical location 24h after Young 1 and its parent initiated migration. Central insert panel: Geographical location of Young 2 and female parent 24h after Young 1 and its parent initiated migration, place of death of Young 2 on 22-Aug 2018.

Panel d: Main panel: Migration tracks of terns belonging to Family unit 4 until death of single young (Young 1) on 20-Aug 2018. Large circles indicate geographical locations of tracked birds when Young 1 and its parent initiated migration, place of death of Young 1 on 20-Aug 2018. Insert panel: Geographical locations of birds when Young 1 and its parent initiated migration.

Panel e: Main panel: Migration tracks of terns belonging to Family unit 5 until failure of Young 1's GPS-tracker on 19-Oct 2018. Large circles indicate geographical locations of tracked birds when Young 1-2 and parental bird initiated migration, tracked birds' geographical location when latter young (Young 2) died & last received GPS-fix location of Young 1 on 19-Oct 2018. Insert panel: The track of

Young 2 during 18-22-Aug 2018 and its geographical location when it died on 22-Aug 2018.

Panel f: Main panel: Migration tracks of terns belonging to Family unit 6 until 31-Dec 2018. Large circles indicate geographical locations of tracked birds when latter young (Young 2) and its parent initiated migration, place of death of Young 1 on 27-Dec 2018. Large (insert) diamonds indicate wintering locations. Insert panel: Geographical locations of birds when Young 2 and its parent initiated migration.

Panel g: Main panel: Migration tracks of terns belonging to Family unit 7 until 31-Dec 2018. Large circles indicate geographical locations of tracked birds when single young (Young 1) and its parent initiated migration, place of death of female parent on 2-Oct 2018. Large diamonds indicate wintering locations. Insert panel: Geographical locations of birds when Young 1 and its parent initiated migration, place of death of female parent on 2-Oct 2018.

Panel h: Migration tracks of terns belonging to Family unit 8 until 31-Dec 2019. Large circles indicate geographical locations of tracked birds when latter young (Young 2) and its parent initiated migration. Large diamonds indicate wintering locations.
