# Supplementary material for: Paternal transmission of migration knowledge in a long-distance bird migrant
Source: Nat Commun. 2022 Mar 23;13:1566. doi: 10.1038/s41467-022-29300-w (PMC8943069; doi:10.1038/s41467-022-29300-w)
Supplement: Supplementary file 3 — Description of Additional Supplementary Files [file 41467_2022_29300_MOESM3_ESM.pdf]

Description of Additional Supplementary Files

File Name: Supplementary Movie 1
Description: Animation of actual biologging data (points seen in animation are GPS-fixes) showing the male parent and young from Family Group 7 (Table S1, Fig. S1) migrating side by side from Pori, Finland to Vormsi, Estonia during 1st -2nd of August 2018
