# Supplementary material for: Paternal transmission of migration knowledge in a long-distance bird migrant
Source: Nat Commun. 2022 Mar 23;13:1566. doi: 10.1038/s41467-022-29300-w (PMC8943069; doi:10.1038/s41467-022-29300-w)
Supplement: Supplementary file 5 — Reporting Summary [file 41467_2022_29300_MOESM5_ESM.pdf]

# nature research

Corresponding author(s): Patrik Byholm

Last updated by author(s): Mar 1, 2022

# Reporting Summary

Nature Research wishes to improve the reproducibility of the work that we publish. This form provides structure for consistency and transparency in reporting. For further information on Nature Research policies, see our Editorial Policies and the Editorial Policy Checklist.

## Statistics

For all statistical analyses, confirm that the following items are present in the figure legend, table legend, main text, or Methods section.

| n/a | Confirmed | |
|---|---|---|
| ☐ | ☒ | The exact sample size (*n*) for each experimental group/condition, given as a discrete number and unit of measurement |
| ☐ | ☒ | A statement on whether measurements were taken from distinct samples or whether the same sample was measured repeatedly |
| ☐ | ☒ | The statistical test(s) used AND whether they are one- or two-sided<br>*Only common tests should be described solely by name; describe more complex techniques in the Methods section.* |
| ☒ | ☐ | A description of all covariates tested |
| ☐ | ☒ | A description of any assumptions or corrections, such as tests of normality and adjustment for multiple comparisons |
| ☐ | ☒ | A full description of the statistical parameters including central tendency (e.g. means) or other basic estimates (e.g. regression coefficient) AND variation (e.g. standard deviation) or associated estimates of uncertainty (e.g. confidence intervals) |
| ☐ | ☒ | For null hypothesis testing, the test statistic (e.g. $F$, $t$, $r$) with confidence intervals, effect sizes, degrees of freedom and $P$ value noted<br>*Give P values as exact values whenever suitable.* |
| ☒ | ☐ | For Bayesian analysis, information on the choice of priors and Markov chain Monte Carlo settings |
| ☒ | ☐ | For hierarchical and complex designs, identification of the appropriate level for tests and full reporting of outcomes |
| ☒ | ☐ | Estimates of effect sizes (e.g. Cohen's *d*, Pearson's *r*), indicating how they were calculated |

*Our web collection on statistics for biologists contains articles on many of the points above.*

## Software and code

Policy information about availability of computer code

| | |
|---|---|
| Data collection | Tracking data of Caspian terns was generated by our team. There was no special software involved. |
| Data analysis | Statistical analyses were conducted in R 4.0.3. using the lme4 and segmented packages, initial handling and sorting of GPS-tracking data as well as construction of maps was performed with ArcMap 10.3.1 and MS Excel 2019. The animated movie in Supplementary Video 1 was made in Google Earth Pro 7.3.3.7786. |

For manuscripts utilizing custom algorithms or software that are central to the research but not yet described in published literature, software must be made available to editors and reviewers. We strongly encourage code deposition in a community repository (e.g. GitHub). See the Nature Research guidelines for submitting code & software for further information.

## Data

Policy information about availability of data

All manuscripts must include a data availability statement. This statement should provide the following information, where applicable:

- Accession codes, unique identifiers, or web links for publicly available datasets
- A list of figures that have associated raw data
- A description of any restrictions on data availability

Metadata on tracked birds is available in Supplementary Table 1. Individual tracking data used in analyses and for constructing Figs. 1-3, Supplementary Fig. 1 and Supplementary Video 1 are available in Movebank under https://doi.org/10.5441/001/1.352qf1cv.

# Field-specific reporting

Please select the one below that is the best fit for your research. If you are not sure, read the appropriate sections before making your selection.

☐ Life sciences ☐ Behavioural & social sciences ☒ Ecological, evolutionary & environmental sciences

For a reference copy of the document with all sections, see nature.com/documents/nr-reporting-summary-flat.pdf

# Ecological, evolutionary & environmental sciences study design

All studies must disclose on these points even when the disclosure is negative.

| | |
|---|---|
| Study description | Statistical and graphical analyses of GPS-tracking device data from Caspian terns (Hydroprogne caspia) breeding in the archipelago of the Baltic Sea to understand how migratory knowledge is socially transmitted from one generation to another in long-distance migratory birds. |
| Research sample | 29 autumn migrations events conducted by 28 wild Caspian terns (n = 13 adults, n = 15 young) from 8 families during 2017-2019 breeding in Western Finland (62°14'N, 21°17'E) were studied (main sample). Birds were tracked using GPS-tracking devices (18 g SAKER-L GPS-GSM tracker, Ecotone (n = 9), 20 g OrniTrack-20, Ornitela (n = 20). Additional autumn migrations events during 2018-2020 made by two adult birds belonging to the sample above (but in different years), two tracks from one new young tagged at the same study site and four tracks from two new young bird tagged in Sweden (57°16'N, 16°37'E and 65°18'N, 22°23'E) were added to the data set on repeat migration analyses (Figure 3; additional sample). Total amount of unique data in this sample not present in the main sample: individuals n = 3, tracks n = 8.<br><br>Blood samples were collected from the brachial vein for determination of sex from DNA. |
| Sampling strategy | Tracking data was obtained for all studied birds. The amount and type of data the devices delivered varied (from 5 min to several hours between GPS-fixes) depending on device model and programming schedule as well as voltage level. Exact number of birds successfully deployed with tracking devices depended on field logistics (access to breeding islets, loss of birds prior to initiation of migration, especially due to predation of naive juveniles by sea eagles (Haliaetus albicilla)). The sample size is nevertheless sufficient for the purpose of the study, i.e to study migration of entire families of birds. |
| Data collection | Adults were caught at the nest during the last week of the incubation phase in late May using spring nets, and juveniles were caught by hand from the breeding islet just prior to fledging. PB, MB, NI and UL were the ones that trapped birds, sampled blood and deployed tracking devices. |
| Timing and spatial scale | Timing: 2017-2020.<br>Spatial scale: The archipelago of the Baltic Sea in Finland and Sweden. |
| Data exclusions | Erroneous GPS-fixes were filtered from the tracking data prior to analyses using a speed filter excluding unrealistic movements between two consecutive fixes. In Fig. 1 and Extended data Fig. 1 the raw tracking data for young birds was down-sampled for better visualisation (retaining all GPS-fixes for both parental birds and young would not allow visualising migration routes of both parties since they are on top of each other since birds migrate together). |
| Reproducibility | An independent data set (different individuals) originating from the same study population presenting information on the migration of adult and young Caspian terns from re-sightings of colour-ringed terns migrating together made by the public during 2016-2020 (family units n = 4, individuals n= 8) confirms the main result, i.e. parental males are the ones responsible for leading young on their first autumn migration as well as the result of re-use of the same migration routes by the same individuals in consecutive years. This data set is available upon request from the authors. |
| Randomization | Not relevant to the present work as it is an observational study. |
| Blinding | Not relevant to the present work as it is an observational study. |

Did the study involve field work? ☒ Yes ☐ No

## Field work, collection and transport

| | |
|---|---|
| Field conditions | In each year during 2017-2019, field work took place during May-August. All breeding islets were reached by boat and island visits lasted for 60-120 minutes. Breeding islets were visited only when it did not rain and the temperature was +15°C to +25°C. |
| Location | Field work was mainly conducted in the Suupohja-archipelago of the Baltic Sea, Western Finland (62°14'N, 21°17'E), but data for one bird tagged at Rödkallen, Northern Sweden (65°18'N, 22°23'E) and another at Furö, Southern Sweden (57°16'N, 16°37'E) were used as part of Figure 3. |
| Access & import/export | All permits needed for field work was obtained from Finnish and Swedish authorities:<br><br>Permits to trap, take blood samples and deploy GPS-tracking devices on Caspian terns in Finland were issued by the Regional State Administrative Agency for Southern Finland (ESAVI/1068/04.10.07/2017) and the Centre for Economic Development, Transport and the Environment in Southwestern Finland (VARELY/875/2017). In Sweden permits were issued by Malmö-Lunds djurförsöksetiska |

| | |
|---|---|
| | nämnd (M470-12, M72-15, M74-20) and by the Swedish Environmental Protection Agency and the Swedish Ringing Office (NV-03567-16).<br><br>To work in protected areas permits were issued by the Centre for Economic Development, Transport and the Environment in Southern Ostrobothnia (EPOELY/1830/215) in Finland and by Norrland County Administration Board (521-4026-18) in Sweden.<br><br>Permit to store biological samples (blood/DNA) for scientific purposes in Finland was issued by and the Centre for Economic Development, Transport and the Environment in Southwestern Finland (VARELY/875/2017). |
| Disturbance | Visits to breeding islets/trapping of birds were done only during periods of good weather and never lasted longer than two hours. No breeding event was disrupted due to research activities. |

# Reporting for specific materials, systems and methods

We require information from authors about some types of materials, experimental systems and methods used in many studies. Here, indicate whether each material, system or method listed is relevant to your study. If you are not sure if a list item applies to your research, read the appropriate section before selecting a response.

## Materials & experimental systems

| n/a | Involved in the study |
|---|---|
| ☒ | ☐ Antibodies |
| ☒ | ☐ Eukaryotic cell lines |
| ☒ | ☐ Palaeontology and archaeology |
| ☐ | ☒ Animals and other organisms |
| ☒ | ☐ Human research participants |
| ☒ | ☐ Clinical data |
| ☒ | ☐ Dual use research of concern |

## Methods

| n/a | Involved in the study |
|---|---|
| ☒ | ☐ ChIP-seq |
| ☒ | ☐ Flow cytometry |
| ☒ | ☐ MRI-based neuroimaging |

## Animals and other organisms

Policy information about studies involving animals; ARRIVE guidelines recommended for reporting animal research

| | |
|---|---|
| Laboratory animals | The study did not involve laboratory animals. |
| Wild animals | All terns were released at the same islet where they were caught (adults: with spring nets, juveniles: by hand prior to fledging) immediately after handling. Details on the number of individuals involved in the study as well as their sex and age is described in the sections above. |
| Field-collected samples | Blood samples (ca. 10-15 µl) collected to sex terns from DNA were put in a blood-buffer and stored at -20 °C in Eppendorf tubes until analyzed. |
| Ethics oversight | All permits needed for trapping, taking blood samples and deploying GPS-tracking devices on Caspian terns, to work in protected areas and to store biological samples (blood/DNA) for scientific purposes were obtained from relevant Finnish and Swedish authorities: (i) the Regional State Administrative Agency for Southern Finland, (ii) the Centre for Economic Development, Transport and the Environment in Southwestern Finland, (iii) the Centre for Economic Development, Transport and the Environment in Southern Ostrobothnia, (iv) Malmö-Lunds djurförsöksetiska nämnd, (v) the Swedish Environmental Protection Agency, (vi) Norrland County Administration Board and (vii) the Swedish Ringing Office. |

Note that full information on the approval of the study protocol must also be provided in the manuscript.
